# Detection of *MYD88 L265P* mutation by next-generation deep sequencing in peripheral blood mononuclear cells of Waldenström’s macroglobulinemia and IgM monoclonal gammopathy of undetermined significance

**DOI:** 10.1371/journal.pone.0221941

**Published:** 2019-09-04

**Authors:** Ayako Nakamura, Chikako Ohwada, Masahiro Takeuchi, Yusuke Takeda, Shokichi Tsukamoto, Naoya Mimura, Oshima-Hasegawa Nagisa, Yasumasa Sugita, Hiroaki Tanaka, Hisashi Wakita, Nobuyuki Aotsuka, Kosei Matsue, Koutaro Yokote, Osamu Ohara, Chiaki Nakaseko, Emiko Sakaida

**Affiliations:** 1 Department of Hematology, Chiba University Hospital, Chiba, Japan; 2 Department of Clinical Cell Biology and Medicine, Chiba University Graduate School of Medicine, Chiba, Japan; 3 Hematology and Oncology, Japanese Red Cross Narita Hospital, Narita, Japan; 4 Department of Transfusion Medicine and Cell Therapy, Chiba University Hospital, Chiba, Japan; 5 Department of Hematology, Oami Municipal Hospital, Oami-Shirasato, Japan; 6 Department of Hematology, Asahi General Hospital, Asahi, Japan; 7 Department of Hematology/Oncology, Kameda General Hospital, Kamogawa, Japan; 8 Department of Technology Development, Kazusa DNA Research Institute, Kisarazu, Japan; 9 Future Medicine Education and Research Organization, Chiba University, Chiba, Japan; 10 Department of Hematology, International University of Health and Welfare School of Medicine, Narita, Japan; European Institute of Oncology, ITALY

## Abstract

We investigated the feasibility of using next-generation sequencing (NGS) technique using molecular barcoding technology to detect *MYD88 L265P* mutation in unselected peripheral blood mononuclear cells (PBMCs) in 52 patients with Waldenström’s macroglobulinemia [1] and 21 patients with IgM-monoclonal gammopathy of undetermined significance (MGUS). The NGS technique successfully detected the *MYD88 L265P* in unselected PBMCs at a sensitivity of 0.02%, which was ×5 higher than that of AS-PCR. All the results between paired BM and PB samples from 2 IgM MGUS and 4 untreated WM patients matched completely. *MYD88 L265P* mutation was detected in 14/21 (66.7%), 14/19 (73.7%), and 10/33 (30.3%) with the median mutant allele burden of 0.36% (range, 0.06–2.85%), 0.48% (range, 0.02–32.3%), and 0.16% (range, 0.02–33.8%), in IgM-MGUS, untreated WM, and previously treated WM, respectively. Multiple linear regression analysis identified an absolute peripheral lymphocyte count as the positive predictor of PB mutant allele burden (R2 = 0,72, P<0.0001). Our non-invasive, simple NGS method has the potential to detect *MYD88 L265P* mutations in PBMCs of IgM MGUS and WM patients, which may especially utilized for monitoring minimal residual tumor burden after treatment.

## Introduction

Waldenström’s Macroglobulinemia [1] is a B-cell malignancy characterized by lymphoplasmacytic cells in bone marrow (BM), lymph nodes and spleen, as well as the abnormal increase in serum immunoglobulin-M (IgM), resulting in many problematic clinical symptoms [2]. In recent years, whole genome sequencing analyses revealed the existence of *MYD88 L265P* somatic mutations, with the presence of 90% of WM patients [3, 4].

*MYD88 L265P* affects the pathophysiology of WM by activating NF-κB in association with interleukin-1 receptor-associated kinase [5] and Bruton’s tyrosine kinase (BTK) [6, 7]. *MYD88 L265P* is associated with a favorable clinical course with a higher treatment response rate to key drugs such as BTK inhibitor (6), which in turn makes its determination increasingly important in a clinical setting. Furthermore, since the majority of IgM monoclonal gammopathy of undetermined significance (MGUS) and a good portion of WM patients may relish asymptomatic phase of the disease [8], a non-invasive method to accurately assess tumor progression is eagerly awaited.

Although recently developed allele-specific polymerase chain reaction (AS-PCR) is highly sensitive in determining the *MYD88 L265P* status and its quantitative assessment may be utilized in monitoring tumor burden [9], CD19-selection technique is required to achieve enough sensitivity when peripheral blood (PB) is used [7], which may not be suitable for clinical use. To provide a more simple, non-invasive, inclusive, as well as sensitive method, we investigated the feasibility of using next-generation sequencing (NGS) technique to detect *MYD88 L265P* from unselected PB mononuclear cells (PBMCs) in WM and IgM-MGUS.

## Patients and methods

This study was approved by the Research Ethics Committee of the Graduate School of Medicine, Chiba University. Patients who visited the participating institutions from February 2017 to December 2017 were recruited, and written informed consent was obtained from all patients. PB was collected from 21 individuals with IgM-MGUS, 19 untreated WM patients, 33 previously treated WM patients, and 5 healthy donors. Paired BM samples are also available in 4 untreated WM and 2 IgM-MGUS patients. All of WM and IgM-MGUS patients met the diagnosis criteria by the World Health Organization classification system. The clinical characteristics of all the patients are described in Table 1.

**Table 1:** Patients’ characteristics at PB sampling.

	IgM MGUS (N = 21) N (%) Median (range)	Untreated WM (N = 19) N (%) Median (range)	Treated WM (N = 33) N (%) Median (range)	*P-* value
Median age (range)	71(50–89)	70 (61–89)	70 (51–87)	0.35
Sex (M:F)	15:6	17:2	25:8	
PS (ECOG) 0–2	20 (95.2)	18 (94.7)	33 (100)	0.19
Adenopathy	0 (0)	2 (10.5)	6 (18.1)	0.69
Symptomatic disease	0 (0)	5 (26.3)	4 (12.5)	0.089
Hemoglobin (g/dL)	13.2 (10.1–16.0)	12.3 (7.6–16.3)	12.4 (7.5–15.6)	0.31
Serum IgM (mg/dL)	742.5 (110–2426)	1425 (140–11940)	1271 (42–9790)	0.14
Serum LDH (mg/dL)	183.5 (103–224)	136 (52–240)	179 (92–267)	0.12
Serum β2MG (mg/dL)	2.0 (1.2–5.3)	2.8 (1.8–7.3)	2.45 (1.4–5.6)	0.059
Platelet count (x10^4^/mm^3^)	23.9 (4.3–51.1)	19.9 (4.2–44.3)	19.5 (2.1–48.1)	0.36
Peripheral lymphocyte count (/mm^3^)	1710 (594–3808)	1620 (836–15075)	1170 (59–5893)	0.015

Genomic DNA was extracted from patients’ PBMCs using a Wizard genomic DNA purification kit (Promega, Madison, WI, USA) and DNA concentration was measured using a NanoDrop Lite (Thermo Scientific, Waltham, MA, USA). 300 ng of DNA was used for the NGS analysis. Variation rates of *MYD88* at the nucleotide position corresponding to L265P were measured with Illumina MiSeq DNA sequencer. Molecular barcoding technology is utilized in order to remove PCR errors and improve precision and accuracy essentially as previously reported [10], with minor modifications as described in the supplementary methods. Quantitative AS-PCR assay was performed with the same samples as previously reported [9]. The sensitivity of NGS and AS-PCR method was determined by serial dilution of the positive mutant control sample with the wild-type DNA of healthy donors.

JMP (SAS Institute Inc., NC, USA) was used to perform the statistical calculations. All tests were two-sided, and a P-value of <0.05 was considered to indicate statistically significant.

## Results

The median coverage of patient samples was 3942 after the error removal. Taken together with serial dilution analysis, our NGS method is capable of detecting *MYD88 L265P* mutation at a sensitivity of 0.02% (Table 2).

**Table 2:** Serial dilution assessment of *MYD88 L265P* mutation.

Dilution factor	1x	4x	20x	100x	200x	500x	1000x	2000x
Estimated value	-	4.828%	0.966%	0.193%	0.097%	0.039%	0.019%	0.010%
Results	19.310%	7.480%	1.510%	0.180%	0.130%	0.060%	0.030%	0.000%

Paired PB and BM samples of 6 patients (2 IgM-MGUS and 4 untreated WM) matched completely in the mutation status between BM and PB by the NGS method, whereas AS-PCR showed inconsistent results in one paired sample (Table 3).

**Table 3:** Paired sample analysis of mononuclear cells obtained from peripheral blood (PB) and bone marrow (BM).

			Bone marrow			Peripheral blood		
			NGS		AS-PCR	NGS		AS-PCR
Age	Sex	Diagnosis	Status	Mutant burden	Status	Status	Mutant burden	Status
59	F	MGUS	Positive	5.87%	Positive	Positive	2.85%	Positive
73	M	MGUS	Positive	1.96%	Positive	Positive	2.30%	Positive
73	M	WM	Positive	2.78%	Positive	Positive	0.23%	Negative
68	M	WM	Positive	0.32%	Positive	Positive	0.14%	Positive
73	M	WM	Positive	24.92%	Positive	Positive	0.27%	Positive
64	M	WM	Negative	0.00%	Negative	Negative	0.00%	Negative

The NGS method detected the mutation with a trend toward higher sensitivity compared to AS-PCR, i.e., 66.7% vs. 42.9% in 21 patients with IgM-MGUS (P = 0.21), 73.7% vs. 56.2% in 19 untreated WM patients (P = 0.32), and a significantly higher sensitivity of 30.3% vs. 9.1% in 33 patients with treated WM (P = 0.03). From the comparison analysis, a significant difference was observed between untreated vs. previously treated WM (73.7% vs. 30.3%, P = 0.01) and IgM-MGUS vs. previously treated WM (66.7% vs. 30.3%, P = 0.02). Among the patients with *MYD88 L265P* mutation, the median percentage of mutant allele relative to wild type was 0.36% in IgM-MGUS (range, 0.06%-2.85%), 0.48% in untreated WM (range, 0.02%–32.3%), and 0.16% in previously treated WM (range, 0.02%-33.8%) with a trend toward lower burden in patients with previously treated WM compared with those with untreated WM (P = 0.14).

Among 31 previously treated WM patients with the response assessment available, 84.8% of patients were treated with rituximab-containing regimens, with an overall response rate of 90% (28/31) as shown in Table 4.

**Table 4:** Treatment regimens and response assessment at the point of PB sampling.

		Treated WM (N = 33) N (%)	
Treatment regimen	Rituximab containing	28	(84.8)
Total number of previous treatment regimens	1	15	(45.4)
	2	11	(33.3)
	> = 3	5	(15.2)
Response assessment at PB sampling	CR	4	(12.1)
	PR	21	(63.6)
	MR	3	(9.1)
	SD+PD	3	(9.1)
	Not evaluated	2	(6.1)

Twenty-eight patients with at least minimal response showed a trend toward lower detection rate of *MYD88 L265P* mutation (25.0% vs. 66.7%, P = 0.19) and mutant allele burden (median, 0.00% vs. 0.22%, P = 0.07), compared to 3 patients less than minimal response.

To identify the disease parameters that correlate with mutant allele burden, we first performed univariate analysis of clinical characteristics at sampling stratified by the *MYD88 L265P* status for all IgM-MGUS and WM patients. Although no significant parameter was identified among IgM-MGUS patients, peripheral lymphocyte count was significantly higher among *MYD88 L265P* positive WM patients compared to the mutation-negative counterpart (Table 5). Multiple linear regression analysis for factors associated with mutant allele burden of PBMCs in WM patients identified absolute peripheral lymphocyte count as the positive predictor of PB mutant allele burden (R^2^ = 0,72, P<0.0001), while serum IgM levels and hemoglobin levels were not selected.

**Table 5:** Univariate analysis of clinical characteristics at sampling stratified by mutation detection status in WM patients.

Variables		Negative		Positive		P-value
		(N = 28) Median (range)		(N = 24) Median (range)		
Age		69	(51–89)	72	(52–86)	0.37
Bence-Jones protein		10/28	(35.1%)	11/24	(45.8%)	0.56
Light chain subtype	Kappa	22	(78.6%)	20	(83.3%)	0.74
	Lambda	6	(21.4%)	4	(16.7%)	
Symptomatic disease		6/28	(21.4%)	3/24	(12.5%)	0.47
Hemoglobin (mg/dL)		12.4	(7.9–16.3)	12.1	(7.5–15.3)	0.85
Serum IgM (mg/dL)		990	(42–9790)	1425	(140–11940)	0.37
Serum β2MG (mg/dL)		2.7	(1.4–5.6)	2.4	(1.8–7.3)	0.93
Platelet count (x10^4^/mm^3^)		18.8	(2.1–48.1)	20.5	(10.7–43.7)	0.29
Peripheral lymphocyte count (/mm^3^)		1170	(59–3463)	1620	(588–15075)	0.007
Bone marrow lymphocytes (%)		26.1	(0.0–72.2)	28.7	(0.0–75.4)	0.75

## Discussion

In this study, the NGS technique using molecular barcoding technology successfully detected the *MYD88 L265P* carried by minimal tumor cells included in unselected PBMCs at a sensitivity of 0.02%. This was ×5 higher than that yielded by AS-PCR [9]. Although the patient number was limited, paired sample analysis of *MYD88 L265P* status in 2 IgM MGUS and 4 previously untreated WM patients showed 100% correlation between BM and PB results, reflecting the high sensitivity and specificity of our NGS assay. Although the direct comparison of NGS and AS-PCR in each disease category revealed a significant statistical advantage only among patients with treated WM, we supposed this is due to the small patient number to prove the advantage of NGS over AS-PCR with enough statistical power.

Notably, our NGS method yielded 66.7% of positivity using unselected PBMCs in IgM-MGUS patients, higher than that of 41.7%, using CD19 sorted PBMCs by AS-PCR in a previous report [7]. At present, there is no reliable method exists for the risk stratification within IgM-MGUS. *MYD88 L265P* status was recently reported to be an independent risk factor in IgM MGUS patients for disease progression to WM [11]. Therefore, our NGS method has a strong potential in carrying out a non-invasive longitudinal progression risk assessment in IgM MGUS patients. Further evaluation is needed to directly compare the two methods by using the same patient samples.

Moreover, molecular barcoding NGS method was able to achieve 73.7% positivity using unselected PBMCs in untreated WM patients, which was higher than the previously reported rate of 40% by AS-PCR [7]. Considering approximately 90–95% of WM patients carry *MYD88 L265P* [4], we still have some room for sensitivity improvement in using unselected PBMCs to determine the mutation status. Increasing the depth of sequencing may be the most effective solution, but its benefit must be balanced with the cost in clinical practice. In the present study, previously treated WM patients showed significantly lower mutation rate and a trend toward lower mutant allele burden, compared to untreated WM and IgM-MGUS. This was consistent with the previous report showing a lower mutation rate and higher delta-Ct in treated WM than in those untreated [7]. We consider that the difference reflects the amount of circulating tumor cells in PB in each disease status. The result of linear regression analysis confirmed our hypothesis by showing that peripheral lymphocyte count was the only factor associated with mutant allele burden, suggesting that NGS is highly capable of detecting minimal circulating tumor cells in PB. This indicates that our NGS method may be useful in assessing treatment response by monitoring tumor burden during patients’ clinical course. Although the current system of treatment response assessment is mainly based on serum IgM levels, frequent discordance between serum IgM and other clinical parameters are observed [12]. In this context, *MYD88* mutant allele frequency yielded by NGS has the potential to provide additional information on disease status assessment. Moreover, since the majority of WM patients undergo treatment with rituximab, as in our study cohort (84.8%), it is essential to include non-B cells like monotypic plasma cells for the accurate assessment of the residual tumor burden and treatment response in previously treated WM [13]. It is noteworthy that our NGS assay use all-inclusive non-selected PBMCs, which gives an advantage upon treatment response assessment in terms of its accuracy over other previously reported CD19 selected methods.

Unlike the previous reports [7], *MYD88 L265P* allele frequency in PBMCs did not correlate with BM tumor burden, serum IgM, and hemoglobin levels in our study cohort. Perhaps because our cohort was too small and heterogeneous, consisting of patients with a wide variety of disease status both with and without treatment.

We used the same amount of DNA (300 ng) all across the samples, regardless of PB lymphocyte count. Increasing the DNA input according to the patients’ lymphocyte counts may expand the coverage, but it must be balanced between the sensitivity and the patients’ physical burden. Recent sensitivity improvements by NGS technology has shown that a very low amount of cancer-associated mutations is detected in normal hematopoietic cells [14]. Although *MYD88* mutation has been recognized as an early oncogenic event in WM, the clinically meaningful threshold of frequency is still unknown. Therefore, the oncogenic mutation with very low frequency must be interpreted with caution. Further improvements will be needed to increase the specificity by optimal threshold setting or combining *MYD88* mutation with other mutation biomarkers, such as *CXCR4*, a next common mutation found in one-third of WM with MYD88 mutation [4].

In conclusion, the NGS technique successfully detected *MYD88 L265P* mutation using unselected PBMCs of WM and IgM MGUS patients with improved sensitivity compared to AS-PCR. Our novel method has the advantage in disease monitoring especially after treatment where inclusion of CD19 negative cells becomes more important. This non-invasive, simple, inclusive NGS method may provide powerful tools to monitor tumor burden during patients’ clinical course, as well as to further investigate the clinical implications of *MYD88 L265P* in IgM MGUS and WM patients.

## Supporting information

S1 File NGS method with molecular barcoding technology. (DOCX)

S2 File Supplementary data set. (CSV)

## References

[1] Mease PJ, Genovese MC, Greenwald MW, Ritchlin CT, Beaulieu AD, Deodhar A, et al Brodalumab, an anti-IL17RA monoclonal antibody, in psoriatic arthritis. The New England journal of medicine. 2014;370(24):2295–306. 10.1056/NEJMoa1315231 . 24918373

[2] Owen RG, Treon SP, Al-Katib A, Fonseca R, Greipp PR, McMaster ML, et al Clinicopathological definition of Waldenstrom’s macroglobulinemia: consensus panel recommendations from the Second International Workshop on Waldenstrom’s Macroglobulinemia. Semin Oncol. 2003;30(2):110–5. 10.1053/sonc.2003.50082 . 12720118

[3] Treon SP, Xu L, Yang G, Zhou Y, Liu X, Cao Y, et al MYD88 L265P somatic mutation in Waldenstrom’s macroglobulinemia. The New England journal of medicine. 2012;367(9):826–33. Epub 2012/08/31. 10.1056/NEJMoa1200710 . 22931316

[4] Treon SP, Cao Y, Xu L, Yang G, Liu X, Hunter ZR. Somatic mutations in MYD88 and CXCR4 are determinants of clinical presentation and overall survival in Waldenstrom macroglobulinemia. Blood. 2014;123(18):2791–6. 10.1182/blood-2014-01-550905 . 24553177

[5] Shimizu M, Kochi T, Shirakami Y, Genovese S, Epifano F, Fiorito S, et al A newly synthesized compound, 4'-geranyloxyferulic acid-N(omega)-nitro-L-arginine methyl ester suppresses inflammation-associated colorectal carcinogenesis in male mice. Int J Cancer. 2014;135(4):774–84. 10.1002/ijc.28718 . 24474144

[6] Lin SC, Lo YC, Wu H. Helical assembly in the MyD88-IRAK4-IRAK2 complex in TLR/IL-1R signalling. Nature. 2010;465(7300):885–90. 10.1038/nature09121 . 20485341 PMC2888693

[7] Xu L, Hunter ZR, Yang G, Cao Y, Liu X, Manning R, et al Detection of MYD88 L265P in peripheral blood of patients with Waldenstrom’s Macroglobulinemia and IgM monoclonal gammopathy of undetermined significance. Leukemia. 2014;28(8):1698–704. 10.1038/leu.2014.65 . 24509637

[8] Kyle RA, Therneau TM, Rajkumar SV, Remstein ED, Offord JR, Larson DR, et al Long-term follow-up of IgM monoclonal gammopathy of undetermined significance. Blood. 2003;102(10):3759–64. 10.1182/blood-2003-03-0801 . 12881316

[9] Xu L, Hunter ZR, Yang G, Zhou Y, Cao Y, Liu X, et al MYD88 L265P in Waldenstrom macroglobulinemia, immunoglobulin M monoclonal gammopathy, and other B-cell lymphoproliferative disorders using conventional and quantitative allele-specific polymerase chain reaction. Blood. 2013;121(11):2051–8. 10.1182/blood-2012-09-454355 . 23321251 PMC3596964

[10] Stahlberg A, Krzyzanowski PM, Egyud M, Filges S, Stein L, Godfrey TE. Simple multiplexed PCR-based barcoding of DNA for ultrasensitive mutation detection by next-generation sequencing. Nat Protoc. 2017;12(4):664–82. 10.1038/nprot.2017.006 . 28253235

[11] Varettoni M, Zibellini S, Arcaini L, Boveri E, Rattotti S, Pascutto C, et al MYD88 (L265P) mutation is an independent risk factor for progression in patients with IgM monoclonal gammopathy of undetermined significance. Blood. 2013;122(13):2284–5. 10.1182/blood-2013-07-513366 . 24072850

[12] Treon SP, Tripsas CK, Meid K, Warren D, Varma G, Green R, et al Ibrutinib in previously treated Waldenstrom’s macroglobulinemia. The New England journal of medicine. 2015;372(15):1430–40. 10.1056/NEJMoa1501548 . 25853747

[13] Gustine J, Meid K, Xu L, Hunter ZR, Castillo JJ, Treon SP. To select or not to select? The role of B-cell selection in determining the MYD88 mutation status in Waldenstrom Macroglobulinaemia. Br J Haematol. 2017;176(5):822–4. 10.1111/bjh.13996 . 26936399

[14] Genovese G, Kahler AK, Handsaker RE, Lindberg J, Rose SA, Bakhoum SF, et al Clonal hematopoiesis and blood-cancer risk inferred from blood DNA sequence. The New England journal of medicine. 2014;371(26):2477–87. 10.1056/NEJMoa1409405 . 25426838 PMC4290021

