# Supplementary material for: Detection of MYD88 L265P mutation by next-generation deep sequencing in peripheral blood mononuclear cells of Waldenström’s macroglobulinemia and IgM monoclonal gammopathy of undetermined significance
Source: PLoS One. 2019 Sep 4;14(9):e0221941. doi: 10.1371/journal.pone.0221941 (PMC6726192; doi:10.1371/journal.pone.0221941)
Supplement: S1 File — (DOCX) [file pone.0221941.s001.docx]

**Supplementary file: NGS method with molecular barcoding technology**

The primer extension was repeated 15 times using a MYD88 L265P-specific primer with a following barcoding sequence;

5’-GGAAACAGCTATGACNNNGNNNNCNNNCAGGTGCCCATCAGAAGC-3’

with a PrimeStar GXL DNA polymerase (Takara Bio). While the 18-nucleotide sequence at the 3’-end of this primer was derived from MYD88 gene, the 15-nucleotide sequence (shown as italic) at the 5’-end was attached for PCR-assisted attachment of Illumina DNA sequencing tags. After removal of the free primers with an Illustra ExoProStar (GE HealthcareLife Science), PCR was conducted with following two primers;

5’-GGAAACAGCTATGAC- 3’

5’-AAAACGACGGCCAGGATGCTGGGGAACTCTTT-3’

PrimeStar GXL DNA polymerase followed by the second-round of PCR for attachment of Illumina DNA sequencing tag containing sample index sequence. After size-selection of PCR products on a BluePippin (3% agarose gel; Sage Science), PCR products were sequenced on MiSeq DNA sequencer with Reagent kit v2. for 300 cycles. Variation rates were calculated by similar procedures as previously reported ^1-3^.

1. Smith T, Heger A, Sudbery I. UMI-tools: modeling sequencing errors in Unique Molecular Identifiers to improve quantification accuracy. *Genome Res.* 2017;27(3):491-499.

2. Stahlberg A, Krzyzanowski PM, Jackson JB, Egyud M, Stein L, Godfrey TE. Simple, multiplexed, PCR-based barcoding of DNA enables sensitive mutation detection in liquid biopsies using sequencing. *Nucleic Acids Res.* 2016;44(11):e105.

3. Masunaga N, Kagara N, Motooka D, et al. Highly sensitive detection of ESR1 mutations in cell-free DNA from patients with metastatic breast cancer using molecular barcode sequencing. *Breast Cancer Res Treat.* 2017.

**Supplementary Table 1.** Serial dilution assessment of MYD88 L265P mutation

| Dilution factor | 1x | 4x | 20x | 100x | 200x | 500x | 1000x | 2000x |
| --- | --- | --- | --- | --- | --- | --- | --- | --- |
| Estimated value | - | 4.828% | 0.966% | 0.193% | 0.097% | 0.039% | 0.019% | 0.010% |
| Results | 19.310% | 7.480% | 1.510% | 0.180% | 0.130% | 0.060% | 0.030% | 0.000% |

**Supplementary table 2.** Paired sample analysis of mononuclear cells obtained from peripheral blood (PB) and bone marrow (BM).

|  |  |  | Bone marrow | | | |  | Peripheral blood | | | |
| --- | --- | --- | --- | --- | --- | --- | --- | --- | --- | --- | --- |
|  |  |  | NGS | |  | AS-PCR |  | NGS | |  | AS-PCR |
| Age | Sex | Diag | Status | Mutant burden |  | Status |  | Status | Mutant burden |  | Status |
| 59 | F | MGUS | Positive | 5.87% |  | Positive |  | Positive | 2.85% |  | Positive |
| 73 | M | MGUS | Positive | 1.96% |  | Positive |  | Positive | 2.30% |  | Positive |
| 73 | M | WM | Positive | 2.78% |  | Positive |  | Positive | 0.23% |  | Negative |
| 68 | M | WM | Positive | 0.32% |  | Positive |  | Positive | 0.14% |  | Positive |
| 73 | M | WM | Positive | 24.92% |  | Positive |  | Positive | 0.27% |  | Positive |
| 64 | M | WM | Negative | 0.00% |  | Negative |  | Negative | 0.00% |  | Negative |

**Supplementary Table 3.** Univariate analysis of clinical characteristics at sampling stratified by mutation detection status in WM patients

| Variables | | Negative | | Positive | | P value |
| --- | --- | --- | --- | --- | --- | --- |
|  |  | (N=28)  Median (range) | | (N=24)  Median (range) | |  |
| Age | | 69 | (51 - 89) | 72 | (52 - 86) | 0.37 |
| Bence-Jones protein | | 10/28 | (35.1%) | 11/24 | (45.8%) | 0.56 |
| Light chain subtype | Kappa | 22 | (78.6%) | 20 | (83.3%) | 0.74 |
|  | Lambda | 6 | (21.4%) | 4 | (16.7%) |  |
| Symptomatic disease | | 6/28 | (21.4%) | 3/24 | (12.5%) | 0.47 |
| Hemoglobin (mg/dL) | | 12.4 | (7.9 - 16.3) | 12.1 | (7.5 -15.3) | 0.85 |
| Serum IgM (mg/dL) | | 990 | (42 - 9790) | 1425 | (140 - 11940) | 0.37 |
| Serum β2MG (mg/dL) | | 2.7 | (1.4 - 5.6) | 2.4 | (1.8 - 7.3) | 0.93 |
| Platelet count (x10^4^/mm^3^) | | 18.8 | (2.1 - 48.1) | 20.5 | (10.7 - 43.7) | 0.29 |
| Peripheral lymphocyte count (/mm^3^) | | 1170 | (59 - 3463) | 1620 | (588 -15075) | 0.007 |
| Bone marrow lymphocytes (%) | | 26.1 | (0.0 - 72.2) | 28.7 | (0.0 - 75.4) | 0.75 |
